# Supplementary material for: Multi‐dataset identification of innovative feature genes and molecular mechanisms in keratoconus
Source: J Cell Mol Med. 2024 Sep 19;28(18):e70079. doi: 10.1111/jcmm.70079 (PMC11412914; doi:10.1111/jcmm.70079)
Supplement: Supplementary file 2 — Material S2. [file JCMM-28-e70079-s002.zip › 7.GSEA analysis/2.ATOH7/KEGG_SPHINGOLIPID_METABOLISM.html]

Details for gene set KEGG\_SPHINGOLIPID\_METABOLISM[GSEA]

|  || Dataset | ATOH7.group.cls#h\_versus\_l |
| Phenotype | group.cls#h\_versus\_l |
| Upregulated in class | h |
| GeneSet | KEGG\_SPHINGOLIPID\_METABOLISM |
| Enrichment Score (ES) | 0.42844936 |
| Normalized Enrichment Score (NES) | 1.4916232 |
| Nominal p-value | 0.04942966 |
| FDR q-value | 0.27437901 |
| FWER p-Value | 0.793 |
Table: GSEA Results Summary

  

Fig 1: Enrichment plot: KEGG\_SPHINGOLIPID\_METABOLISM      
 Profile of the Running ES Score & Positions of GeneSet Members on the Rank Ordered List

  

| SYMBOL | TITLE | RANK IN GENE LIST | RANK METRIC SCORE | RUNNING ES | CORE ENRICHMENT || 1 | SGPP2 | na | 883 | 0.466 | 0.0931 | Yes |
| 2 | DEGS1 | na | 2730 | 0.297 | 0.1153 | Yes |
| 3 | NEU2 | na | 2782 | 0.293 | 0.1884 | Yes |
| 4 | GAL3ST1 | na | 3320 | 0.264 | 0.2401 | Yes |
| 5 | UGCG | na | 3815 | 0.243 | 0.2877 | Yes |
| 6 | SMPD3 | na | 4923 | 0.204 | 0.3077 | Yes |
| 7 | NEU3 | na | 5142 | 0.196 | 0.3513 | Yes |
| 8 | SGMS2 | na | 5499 | 0.185 | 0.3882 | Yes |
| 9 | SMPD1 | na | 6446 | 0.158 | 0.4011 | Yes |
| 10 | SMPD2 | na | 7666 | 0.126 | 0.3981 | Yes |
| 11 | ACER1 | na | 7843 | 0.122 | 0.4240 | Yes |
| 12 | CERK | na | 8626 | 0.106 | 0.4284 | Yes |
| 13 | SPHK2 | na | 9717 | 0.086 | 0.4189 | No |
| 14 | ASAH2 | na | 10517 | 0.073 | 0.4145 | No |
| 15 | SPTLC1 | na | 11286 | 0.062 | 0.4081 | No |
| 16 | GLB1 | na | 11405 | 0.060 | 0.4201 | No |
| 17 | ASAH1 | na | 13296 | 0.037 | 0.3751 | No |
| 18 | DEGS2 | na | 13305 | 0.037 | 0.3844 | No |
| 19 | NEU1 | na | 13723 | 0.033 | 0.3808 | No |
| 20 | SGMS1 | na | 13935 | 0.031 | 0.3826 | No |
| 21 | GLA | na | 13992 | 0.031 | 0.3888 | No |
| 22 | ARSA | na | 14872 | 0.022 | 0.3691 | No |
| 23 | SPTLC2 | na | 15741 | 0.015 | 0.3479 | No |
| 24 | SMPD4 | na | 18734 | -0.011 | 0.2643 | No |
| 25 | NEU4 | na | 20114 | -0.023 | 0.2304 | No |
| 26 | KDSR | na | 21093 | -0.033 | 0.2105 | No |
| 27 | ACER3 | na | 22096 | -0.044 | 0.1928 | No |
| 28 | SPHK1 | na | 23089 | -0.056 | 0.1784 | No |
| 29 | ENPP7 | na | 23340 | -0.059 | 0.1863 | No |
| 30 | SGPP1 | na | 23605 | -0.064 | 0.1950 | No |
| 31 | ACER2 | na | 24471 | -0.077 | 0.1895 | No |
| 32 | GALC | na | 25019 | -0.086 | 0.1955 | No |
| 33 | SGPL1 | na | 25533 | -0.095 | 0.2049 | No |
| 34 | B4GALT6 | na | 26290 | -0.111 | 0.2112 | No |
| 35 | UGT8 | na | 27015 | -0.126 | 0.2223 | No |
Table: GSEA details [plain text format]

  

Fig 2: KEGG\_SPHINGOLIPID\_METABOLISM      
 Blue-Pink O' Gram in the Space of the Analyzed GeneSet

  

Fig 3: KEGG\_SPHINGOLIPID\_METABOLISM: Random ES distribution      
 Gene set null distribution of ES for **KEGG\_SPHINGOLIPID\_METABOLISM**
